# Hexa­kis­(μ_3_-2-hy­droxy­naphthalene-1-carboxaldehyde thio­semicarbazonato-κ^3^ *N* ^2^:*S*:*S*)hexa­silver(I) *N*,*N*-dimethyl­formamide tetra­solvate

**DOI:** 10.1107/S1600536812050155

**Published:** 2012-12-15

**Authors:** Qiaozhen Sun, Liyuan Chai, Hui Liu, Junke Wang

**Affiliations:** a Department of Materials Chemistry, School of Materials Science and Engineering, Key Laboratory of Nonferrous Metal of the Ministry of Education, Central South University, Changsha 410083, People’s Republic of China; b Chinese National Engineering Research Center for Control & Treatment of Heavy Metal Pollution (CNERC–CTHMP), Environmental Engineering Institute, School of Metallurgical Science and Engineering, Central South University, Changsha 410083, People’s Republic of China

## Abstract

In the title compound, [Ag_6_(C_12_H_10_N_3_OS)_6_]·4C_3_H_7_NO, the hexa­nuclear complex mol­ecule lies about an inversion center. The six Ag atoms form a distorted octa­hedron, with Ag⋯Ag distances in the range 2.933 (1)–3.401 (1) Å. Each Ag atom is surrounded by one N atom and two thiol­ate S atoms from two deprotonated 2-hy­droxy-1-naphthaldehyde thio­semi­carb­a­zone ligands. Each ligand coordinates three Ag atoms *via* a bridging thiol­ate S atom and a monodentate N atom, thus two Ag_3_S_3_ hexa­gonal rings are linked together. Two dimethyl­formamide solvent mol­ecules are located in four sets of sites with half-occupancy and form O⋯H—N hydrogen bonds to the complex mol­ecule. Intra­molecular O—H⋯N hydrogen bonds are also present. The discrete hexa­nuclear clusters are further linked through π–π inter­actions into layers parallel to (001), the shortest distance between the centroids of aromatic rings being 3.698 (2) Å.

## Related literature

For the structure and luminescent properties of *d* ^10^ metal complexes, see: Brito *et al.* (2011 ▶); Forward *et al.* (1995 ▶). For structures of related compexes with thio­semicarbazone Schiff base ligands, see: Ashfield *et al.* (2004 ▶); Castiñeiras & Pedrido (2009 ▶); Li *et al.* (2010 ▶); Onodera *et al.* (2007 ▶); Pedrido *et al.* (2009 ▶); Sun (2011 ▶); Sun *et al.* (2012 ▶); Sun & Chai (2012 ▶); Xu *et al.* (2011 ▶). For bond-length data, see: Han *et al.* (2004 ▶).
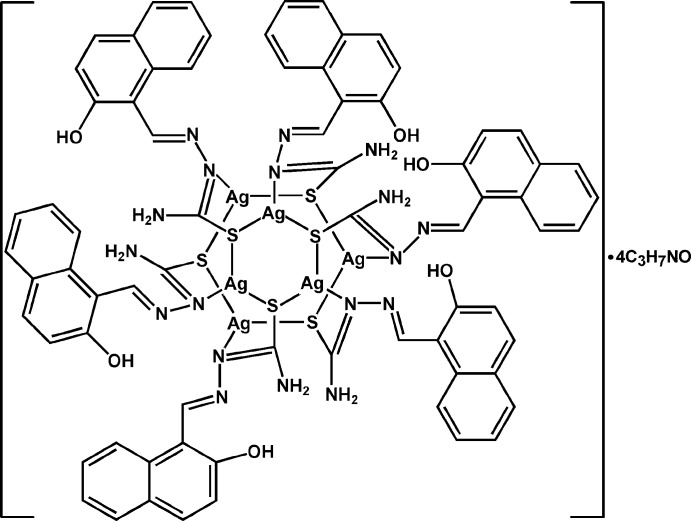


## Experimental

### 

#### Crystal data

[Ag_6_(C_12_H_10_N_3_OS)_6_]·4C_3_H_7_NO
*M* *_r_* = 2405.34
Monoclinic, 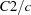

*a* = 24.604 (3) Å
*b* = 18.877 (3) Å
*c* = 24.816 (3) Å
β = 94.763 (3)°
*V* = 11486 (3) Å^3^
*Z* = 4
Mo *K*α radiation
μ = 1.17 mm^−1^
*T* = 293 K
0.22 × 0.20 × 0.18 mm

#### Data collection

Bruker SMART CCD diffractometer
Absorption correction: multi-scan (*SADABS*; Sheldrick, 1996 ▶) *T*_min_ = 0.238, *T*_max_ = 0.373
28454 measured reflections
10056 independent reflections
7829 reflections with *I* > 2s(*I*)
*R*_int_ = 0.042

#### Refinement

*R*[*F* ^2^ > 2σ(*F* ^2^)] = 0.046
*wR*(*F* ^2^) = 0.145
*S* = 1.08
10056 reflections
667 parameters
63 restraints
H-atom parameters constrained
Δρ_max_ = 0.92 e Å^−3^
Δρ_min_ = −0.42 e Å^−3^

### 

Data collection: *SMART* (Bruker, 2000 ▶); cell refinement: *SAINT* (Bruker, 2000 ▶); data reduction: *SAINT*; program(s) used to solve structure: *SHELXTL* (Sheldrick, 2008 ▶); program(s) used to refine structure: *SHELXTL*; molecular graphics: *SHELXTL*; software used to prepare material for publication: *SHELXTL*.

## Supplementary Material

Crystal structure: contains datablock(s) global, I. DOI: 10.1107/S1600536812050155/yk2079sup1.cif

Structure factors: contains datablock(s) I. DOI: 10.1107/S1600536812050155/yk2079Isup2.hkl

Additional supplementary materials:  crystallographic information; 3D view; checkCIF report

## Figures and Tables

**Table 1:** Hydrogen-bond geometry (Å, °)

*D*—H⋯*A*	*D*—H	H⋯*A*	*D*⋯*A*	*D*—H⋯*A*
N3—H3*B*⋯O4	0.86	1.98	2.835 (8)	175
N6—H6*B*⋯O6^i^	0.86	2.00	2.845 (7)	166
N9—H9*A*⋯O5^ii^	0.86	2.31	3.049 (8)	145
N9—H9*B*⋯O7	0.86	2.02	2.870 (6)	172
O1—H1*B*⋯N1	0.82	1.86	2.588 (5)	147
O2—H2*B*⋯N4	0.82	1.85	2.583 (4)	148
O3—H3*C*⋯N7	0.82	1.86	2.587 (5)	147

Symmetry codes: (i) 
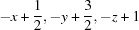
; (ii) 
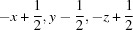
.

**Fig. 1.**
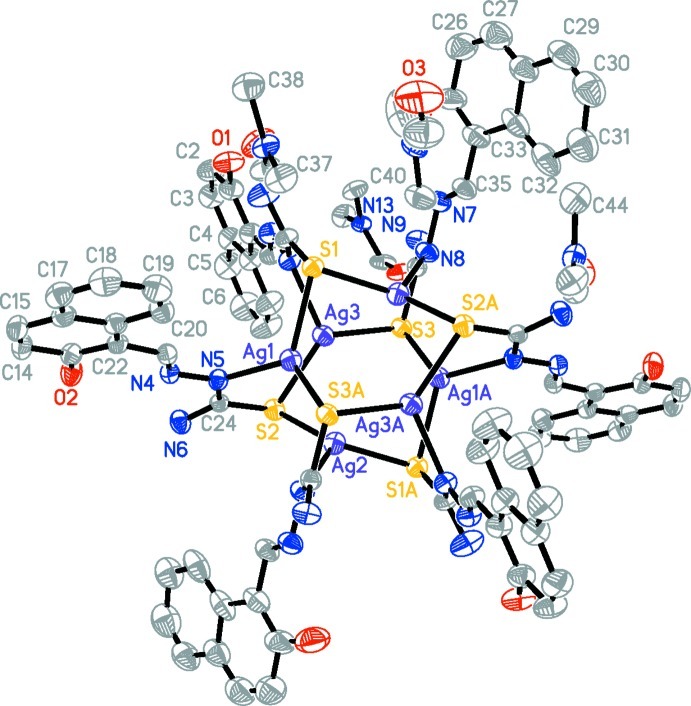
The structure of title compound showing the atom-numbering scheme with H atoms omitted for clarity. Displacement ellipsoids are drawn at the 30% probability level. [Symmetry code: (i) -x + 0.5, -y + 1.5, -z + 2]

**Fig. 2.**
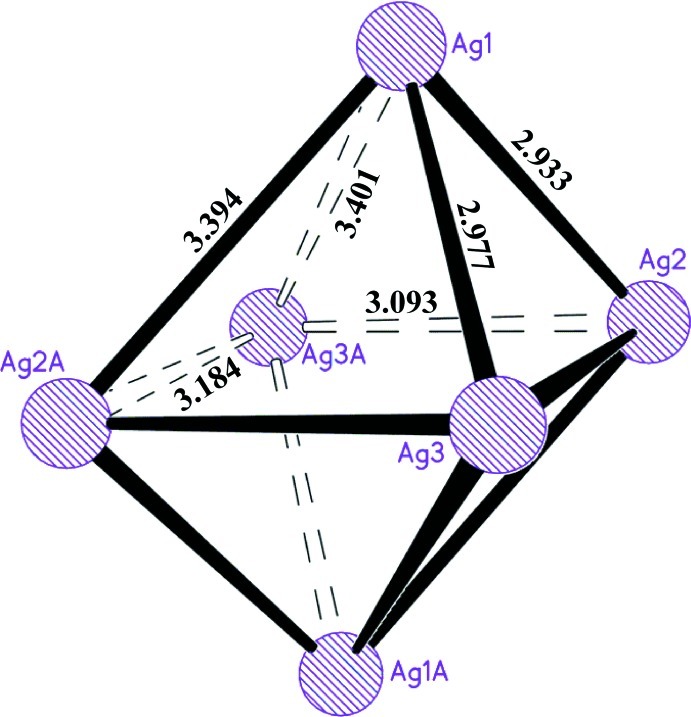
Ag6 octahedron in the title compound

**Fig. 3.**
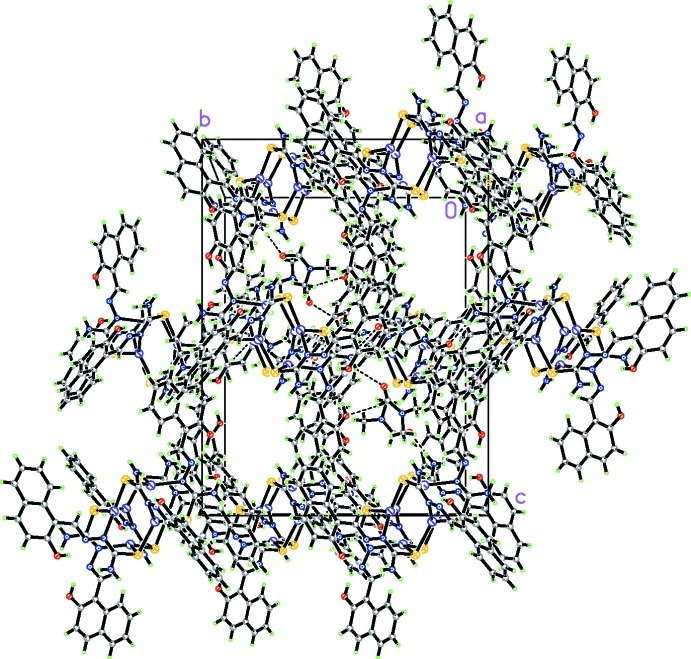
Packing diagram of the title compound, viewed along the a axis direction.

## supplementary crystallographic information

### Comment

Transition metal-chalcogen compounds, especially for *d*^10^ metal complexes, have attracted a great deal of attention for their interesting structures and excellent luminescent properties (Brito *et al.*, 2011; Forward *et al.*, 1995). Of which many coordination complexes with thiosemicarbazone Schiff base ligands have been reported (Ashfield *et al.*, 2004; Castiñeiras & Pedrido, 2009; Li *et al.*, 2010; Onodera *et al.*, 2007; Pedrido *et al.*, 2009). As a part of our studies on this class of compounds (Sun, 2011; Sun *et al.*, 2012; Sun & Chai, 2012; Xu *et al.*, 2011), we describe here the structure of the title compound.

The structure of the title compound is shown in Fig. 1. It contains an Ag_6_ hexanuclear cluster with the Ag···Ag distances varying from 2.93 Å to 3.40 Å (Fig. 2), which is shorter than the sum of van der Waals radii of two silver atoms (3.44 Å) (Han *et al.*, 2004). In the cluster, each Ag(I) ion is surrounded by one nitrogen atom and two thiolate sulfur atoms from two deprotonated ligands *L*^5^. Each ligand coordinates to three Ag(I) ions using a bridged thiolate sulfur atom and a monodentate nitrogen atom, from which two Ag_3_S_3_ hexagonal rings are linked together to give the overall Ferris wheel structure.

There are intramolecular hydrogen bonds of O—H···N type. Besides this, solvent DMF molecules are linked to the hexanuclear cluster *via* O···H—N hydrogen bonds.

Packing of the title compound (Fig. 3) is facilitated through π–π stacking interactions between aromatic rings I, II [defined by the atoms C(1), C(2), C(3), C(4), C(9) and C(10) and the atoms C(13), C(14), C(15), C(16), C(21) and C(22), respectively] and the symmetry related ones (ring centroid distances: 3.78 Å and 3.70 Å, respectively).

### Experimental

Triethylamine (25 µL, 0.175 mmol) was added to a solution of *L*^5^ (0.175 mmol, 0.043 g) in 3 ml DMF. After stirring for 30 min, a DMF solution (2 ml) of AgNO_3_ (0.175 mmol, 0.030 g) was added. Block yellow crystals were formed by standing the solution in air for two months. Anal. Calcd for C_84_H_88_Ag_6_N_22_O_10_S_6_: C, 41.9; H, 3.7; N, 12.8. Found: C, 41.9; H, 3.6; N, 12.8.

### Refinement

In the compound, all the DMF molecules were found to be disordered, and the s.o.f. for the four disordered molecules were fixed at 0.5. All of the non-hydrogen atoms were refined with anisotropic thermal displacement parameters. The H atoms were placed in calculated positions using the riding model approximation with C—H distances of 0.93–0.96 Å, O—H distances of 0.82 Å and N—H distances of 0.86 Å. *U*_iso_(H) were set to 1.2Ueq (C, N) or 1.5Ueq(C, O).

### Crystal data

**Table:**

[Ag_6_(C_12_H_10_N_3_OS)_6_]·4C_3_H_7_NO	*F*(000) = 4816
*M**_r_* = 2405.34	*D*_x_ = 1.391 Mg m^−^^3^
Monoclinic, *C*2/*c*	Mo *K*α radiation, λ = 0.71073 Å
Hall symbol: -C 2yc	Cell parameters from 6309 reflections
*a* = 24.604 (3) Å	θ = 2.2–27.2°
*b* = 18.877 (3) Å	µ = 1.17 mm^−^^1^
*c* = 24.816 (3) Å	*T* = 293 K
β = 94.763 (3)°	Block, yellow
*V* = 11486 (3) Å^3^	0.22 × 0.20 × 0.18 mm
*Z* = 4	

### Data collection

**Table:**

Bruker SMART CCD diffractometer	10056 independent reflections
Radiation source: fine-focus sealed tube	7829 reflections with *I* > 2s(*I*)
Graphite monochromator	*R*_int_ = 0.042
φ and ω scans	θ_max_ = 25.0°, θ_min_ = 1.7°
Absorption correction: multi-scan (*SADABS*; Sheldrick, 1996)	*h* = −28→29
*T*_min_ = 0.238, *T*_max_ = 0.373	*k* = −22→21
28454 measured reflections	*l* = −29→16

### Refinement

**Table:**

Refinement on *F*^2^	Primary atom site location: structure-invariant direct methods
Least-squares matrix: full	Secondary atom site location: difference Fourier map
*R*[*F*^2^ > 2σ(*F*^2^)] = 0.046	Hydrogen site location: inferred from neighbouring sites
*wR*(*F*^2^) = 0.145	H-atom parameters constrained
*S* = 1.08	*w* = 1/[σ^2^(*F*_o_^2^) + (0.095*P*)^2^] where *P* = (*F*_o_^2^ + 2*F*_c_^2^)/3
10056 reflections	(Δ/σ)_max_ = 0.002
667 parameters	Δρ_max_ = 0.92 e Å^−^^3^
63 restraints	Δρ_min_ = −0.42 e Å^−^^3^

### Special details

**Table:**

Geometry. All e.s.d.'s (except the e.s.d. in the dihedral angle between two l.s. planes) are estimated using the full covariance matrix. The cell e.s.d.'s are taken into account individually in the estimation of e.s.d.'s in distances, angles and torsion angles; correlations between e.s.d.'s in cell parameters are only used when they are defined by crystal symmetry. An approximate (isotropic) treatment of cell e.s.d.'s is used for estimating e.s.d.'s involving l.s. planes.
Refinement. Refinement of *F*^2^ against ALL reflections. The weighted *R*-factor *wR* and goodness of fit *S* are based on *F*^2^, conventional *R*-factors *R* are based on *F*, with *F* set to zero for negative *F*^2^. The threshold expression of *F*^2^ > σ(*F*^2^) is used only for calculating *R*-factors(gt) *etc*. and is not relevant to the choice of reflections for refinement. *R*-factors based on *F*^2^ are statistically about twice as large as those based on *F*, and *R*- factors based on ALL data will be even larger.

### Fractional atomic coordinates and isotropic or equivalent isotropic displacement parameters (Å^2^)

**Table:**

	*x*	*y*	*z*	*U*_iso_*/*U*_eq_	Occ. (<1)
Ag1	0.171626 (12)	0.813538 (16)	0.482106 (12)	0.05265 (8)	
Ag2	0.240712 (12)	0.788788 (17)	0.582419 (12)	0.05635 (9)	
Ag3	0.191030 (13)	0.661685 (16)	0.509778 (12)	0.05414 (8)	
S1	0.16540 (4)	0.75257 (5)	0.39220 (4)	0.0510 (2)	
S2	0.16228 (4)	0.70906 (5)	0.59592 (4)	0.0520 (2)	
S3	0.27035 (4)	0.57841 (5)	0.51287 (4)	0.0499 (2)	
N1	0.09822 (14)	0.57526 (18)	0.43441 (14)	0.0591 (9)	
N2	0.13114 (13)	0.63483 (17)	0.43751 (13)	0.0531 (8)	
N3	0.08875 (19)	0.6661 (3)	0.35352 (18)	0.0993 (14)	
H3A	0.0673	0.6301	0.3527	0.119*	
H3B	0.0868	0.6956	0.3271	0.119*	
N4	0.05033 (12)	0.83205 (16)	0.52337 (13)	0.0475 (8)	
N5	0.10008 (12)	0.79779 (16)	0.53377 (12)	0.0476 (8)	
N6	0.06729 (14)	0.76102 (19)	0.61488 (13)	0.0614 (9)	
H6A	0.0380	0.7858	0.6092	0.074*	
H6B	0.0725	0.7360	0.6438	0.074*	
N7	0.27039 (15)	0.5619 (2)	0.35485 (14)	0.0656 (10)	
N8	0.27440 (14)	0.59230 (18)	0.40692 (12)	0.0575 (9)	
N9	0.25925 (15)	0.47922 (19)	0.43895 (15)	0.0671 (10)	
H9A	0.2586	0.4610	0.4071	0.080*	
H9B	0.2547	0.4527	0.4664	0.080*	
O1	0.02497 (15)	0.48899 (18)	0.39190 (13)	0.0853 (10)	
H1B	0.0463	0.5224	0.3930	0.128*	
O2	−0.05009 (11)	0.86290 (17)	0.53609 (12)	0.0686 (8)	
H2B	−0.0209	0.8420	0.5413	0.103*	
O3	0.2174 (2)	0.4973 (3)	0.27494 (18)	0.1230 (16)	
H3C	0.2245	0.5096	0.3064	0.184*	
C1	0.0316 (2)	0.4524 (2)	0.4389 (2)	0.0696 (12)	
C2	−0.0022 (2)	0.3926 (3)	0.4433 (2)	0.0858 (16)	
H2A	−0.0264	0.3788	0.4144	0.103*	
C3	0.0012 (2)	0.3550 (3)	0.4907 (2)	0.0824 (15)	
H3D	−0.0213	0.3157	0.4936	0.099*	
C4	0.0369 (2)	0.3736 (2)	0.5340 (2)	0.0710 (13)	
C5	0.0388 (3)	0.3357 (3)	0.5855 (3)	0.0961 (18)	
H5A	0.0155	0.2975	0.5892	0.115*	
C6	0.0736 (3)	0.3551 (3)	0.6272 (3)	0.109 (2)	
H6C	0.0739	0.3303	0.6596	0.131*	
C7	0.1083 (3)	0.4100 (3)	0.6233 (2)	0.104 (2)	
H7B	0.1324	0.4220	0.6527	0.125*	
C8	0.1084 (2)	0.4484 (3)	0.5761 (2)	0.0873 (16)	
H8B	0.1330	0.4856	0.5742	0.105*	
C9	0.07184 (19)	0.4329 (2)	0.53019 (19)	0.0678 (12)	
C10	0.06846 (18)	0.4728 (2)	0.48118 (18)	0.0633 (11)	
C11	0.10170 (17)	0.5363 (2)	0.47600 (18)	0.0600 (11)	
H11A	0.1266	0.5487	0.5047	0.072*	
C12	0.12505 (16)	0.6761 (2)	0.39585 (16)	0.0529 (10)	
C13	−0.04809 (15)	0.9071 (2)	0.49380 (17)	0.0547 (10)	
C14	−0.09473 (17)	0.9478 (2)	0.47924 (19)	0.0659 (12)	
H14A	−0.1254	0.9428	0.4984	0.079*	
C15	−0.09576 (17)	0.9941 (3)	0.4380 (2)	0.0671 (12)	
H15A	−0.1271	1.0207	0.4294	0.080*	
C16	−0.05039 (17)	1.0030 (2)	0.40773 (17)	0.0602 (11)	
C17	−0.0514 (2)	1.0516 (3)	0.36387 (19)	0.0711 (13)	
H17A	−0.0823	1.0790	0.3556	0.085*	
C18	−0.0087 (2)	1.0587 (3)	0.3344 (2)	0.0818 (15)	
H18A	−0.0103	1.0906	0.3058	0.098*	
C19	0.0376 (2)	1.0188 (3)	0.3463 (2)	0.0780 (14)	
H19A	0.0667	1.0235	0.3249	0.094*	
C20	0.04169 (19)	0.9720 (2)	0.38905 (18)	0.0654 (12)	
H20A	0.0739	0.9468	0.3968	0.078*	
C21	−0.00225 (15)	0.9620 (2)	0.42120 (16)	0.0521 (10)	
C22	−0.00177 (15)	0.9137 (2)	0.46551 (15)	0.0497 (9)	
C23	0.04720 (15)	0.87334 (19)	0.48270 (15)	0.0484 (9)	
H23A	0.0776	0.8780	0.4630	0.058*	
C24	0.10444 (14)	0.76144 (19)	0.57889 (14)	0.0435 (8)	
C25	0.2510 (3)	0.5314 (4)	0.2427 (2)	0.104 (2)	
C26	0.2459 (3)	0.5160 (4)	0.1857 (3)	0.1147 (18)	
H26A	0.2203	0.4836	0.1712	0.138*	
C27	0.2789 (3)	0.5496 (4)	0.1544 (3)	0.1144 (18)	
H27A	0.2745	0.5391	0.1177	0.137*	
C28	0.3180 (3)	0.5973 (3)	0.1699 (2)	0.0934 (15)	
C29	0.3531 (3)	0.6318 (4)	0.1345 (2)	0.1046 (18)	
H29A	0.3493	0.6213	0.0977	0.126*	
C30	0.3886 (3)	0.6755 (4)	0.1515 (3)	0.127 (2)	
H30A	0.4086	0.6988	0.1268	0.152*	
C31	0.3996 (3)	0.6910 (4)	0.2081 (3)	0.114 (2)	
H31A	0.4273	0.7223	0.2199	0.137*	
C32	0.3687 (3)	0.6590 (3)	0.2442 (2)	0.1015 (19)	
H32A	0.3757	0.6680	0.2809	0.122*	
C33	0.3264 (2)	0.6125 (3)	0.2266 (2)	0.0911 (16)	
C34	0.2898 (2)	0.5779 (3)	0.26316 (19)	0.0814 (15)	
C35	0.29397 (18)	0.5973 (3)	0.32006 (16)	0.0645 (12)	
H35A	0.3145	0.6368	0.3313	0.077*	
C36	0.26717 (15)	0.5477 (2)	0.44566 (15)	0.0488 (9)	
C37	0.1231 (5)	0.9297 (5)	0.2325 (5)	0.087 (3)	0.50
H37A	0.1419	0.9325	0.2679	0.130*	0.50
H37B	0.0973	0.9678	0.2277	0.130*	0.50
H37C	0.1489	0.9331	0.2056	0.130*	0.50
C38	0.0619 (5)	0.8437 (6)	0.1746 (5)	0.089 (3)	0.50
H38A	0.0455	0.7980	0.1785	0.133*	0.50
H38B	0.0854	0.8423	0.1456	0.133*	0.50
H38C	0.0338	0.8784	0.1667	0.133*	0.50
C39	0.0960 (4)	0.8193 (6)	0.2672 (4)	0.078 (3)	0.50
H39A	0.1113	0.8342	0.3008	0.093*	0.50
N10	0.0949 (3)	0.8634 (3)	0.2267 (3)	0.0561 (17)	0.50
O4	0.0773 (3)	0.7578 (4)	0.2628 (3)	0.095 (2)	0.50
C40	0.2559 (5)	0.7879 (6)	0.2711 (4)	0.087 (3)	0.50
H40A	0.2754	0.8308	0.2806	0.130*	0.50
H40B	0.2801	0.7482	0.2767	0.130*	0.50
H40C	0.2260	0.7828	0.2932	0.130*	0.50
C41	0.2129 (6)	0.7401 (7)	0.2012 (6)	0.161 (6)	0.50
H41A	0.2007	0.7454	0.1636	0.242*	0.50
H41B	0.1820	0.7328	0.2217	0.242*	0.50
H41C	0.2369	0.7001	0.2057	0.242*	0.50
C42	0.2428 (7)	0.8450 (8)	0.1807 (6)	0.125 (5)	0.50
H42A	0.2656	0.8799	0.1965	0.150*	0.50
N11	0.2359 (3)	0.7908 (4)	0.2164 (3)	0.0563 (18)	0.50
O5	0.2296 (5)	0.8580 (5)	0.1406 (3)	0.136 (4)	0.50
O6	0.4334 (3)	0.8328 (4)	0.2955 (3)	0.090 (2)	0.50
C43	0.4240 (4)	1.0056 (5)	0.2406 (4)	0.081 (3)	0.50
H43A	0.4350	1.0254	0.2754	0.121*	0.50
H43B	0.3889	1.0239	0.2279	0.121*	0.50
H43C	0.4502	1.0180	0.2155	0.121*	0.50
C44	0.4052 (4)	0.8921 (6)	0.1971 (4)	0.084 (3)	0.50
H44A	0.4048	0.8424	0.2051	0.126*	0.50
H44B	0.4307	0.9012	0.1707	0.126*	0.50
H44C	0.3694	0.9069	0.1832	0.126*	0.50
C45	0.4319 (4)	0.8969 (7)	0.2912 (5)	0.089 (3)	0.50
H45A	0.4389	0.9239	0.3224	0.107*	0.50
N12	0.4210 (3)	0.9304 (4)	0.2449 (2)	0.063 (2)	0.50
O7	0.2454 (2)	0.3792 (3)	0.5239 (2)	0.0587 (13)	0.50
C46	0.1547 (4)	0.2320 (4)	0.5242 (4)	0.075 (3)	0.50
H46A	0.1703	0.2201	0.5598	0.112*	0.50
H46B	0.1600	0.1934	0.5000	0.112*	0.50
H46C	0.1164	0.2410	0.5252	0.112*	0.50
C47	0.1648 (4)	0.3228 (5)	0.4527 (4)	0.073 (3)	0.50
H47A	0.1858	0.3643	0.4461	0.109*	0.50
H47B	0.1268	0.3351	0.4510	0.109*	0.50
H47C	0.1704	0.2876	0.4259	0.109*	0.50
C48	0.2207 (4)	0.3293 (4)	0.5356 (3)	0.060 (2)	0.50
H48A	0.2294	0.3111	0.5701	0.072*	0.50
N13	0.1815 (3)	0.2954 (3)	0.5052 (3)	0.0468 (15)	0.50

### Atomic displacement parameters (Å^2^)

**Table:**

	*U*^11^	*U*^22^	*U*^33^	*U*^12^	*U*^13^	*U*^23^
Ag1	0.05776 (17)	0.05486 (17)	0.04597 (16)	−0.00022 (13)	0.00805 (13)	0.00734 (12)
Ag2	0.05578 (17)	0.06027 (18)	0.05285 (18)	0.00050 (13)	0.00367 (14)	0.00891 (13)
Ag3	0.06226 (18)	0.05244 (17)	0.04791 (17)	0.00372 (13)	0.00561 (14)	0.00294 (12)
S1	0.0549 (5)	0.0543 (5)	0.0438 (5)	−0.0023 (4)	0.0035 (4)	0.0061 (4)
S2	0.0564 (5)	0.0552 (5)	0.0450 (5)	0.0023 (4)	0.0079 (4)	0.0091 (4)
S3	0.0570 (5)	0.0488 (5)	0.0446 (5)	0.0013 (4)	0.0081 (4)	0.0031 (4)
N1	0.0648 (19)	0.0566 (19)	0.0556 (19)	−0.0095 (16)	0.0042 (16)	0.0038 (16)
N2	0.0554 (17)	0.0538 (18)	0.0497 (18)	−0.0064 (15)	0.0017 (14)	0.0065 (15)
N3	0.109 (3)	0.098 (3)	0.085 (3)	−0.033 (2)	−0.027 (2)	0.016 (2)
N4	0.0439 (15)	0.0487 (17)	0.0504 (17)	0.0009 (13)	0.0069 (13)	0.0001 (14)
N5	0.0439 (15)	0.0514 (17)	0.0480 (17)	0.0004 (13)	0.0064 (13)	0.0060 (13)
N6	0.0601 (19)	0.073 (2)	0.0532 (19)	0.0072 (17)	0.0165 (16)	0.0100 (16)
N7	0.079 (2)	0.068 (2)	0.0490 (19)	0.0157 (18)	0.0009 (17)	−0.0046 (17)
N8	0.071 (2)	0.063 (2)	0.0390 (17)	0.0076 (17)	0.0063 (15)	0.0032 (15)
N9	0.091 (3)	0.057 (2)	0.054 (2)	−0.0044 (18)	0.0069 (18)	−0.0029 (16)
O1	0.116 (3)	0.073 (2)	0.0638 (19)	−0.0227 (19)	−0.0104 (18)	−0.0023 (16)
O2	0.0522 (15)	0.084 (2)	0.0713 (19)	0.0015 (15)	0.0128 (14)	0.0191 (16)
O3	0.131 (3)	0.137 (4)	0.095 (3)	−0.006 (3)	−0.029 (3)	−0.024 (3)
C1	0.082 (3)	0.057 (2)	0.070 (3)	−0.012 (2)	0.007 (2)	−0.005 (2)
C2	0.105 (4)	0.064 (3)	0.086 (4)	−0.028 (3)	−0.005 (3)	−0.007 (3)
C3	0.093 (3)	0.056 (3)	0.099 (4)	−0.024 (2)	0.011 (3)	−0.001 (3)
C4	0.081 (3)	0.053 (2)	0.080 (3)	−0.011 (2)	0.012 (2)	0.006 (2)
C5	0.110 (4)	0.067 (3)	0.112 (5)	−0.018 (3)	0.013 (4)	0.020 (3)
C6	0.135 (5)	0.097 (4)	0.095 (4)	−0.015 (4)	0.008 (4)	0.041 (3)
C7	0.127 (5)	0.093 (4)	0.088 (4)	−0.028 (4)	−0.022 (3)	0.032 (3)
C8	0.093 (3)	0.075 (3)	0.091 (4)	−0.023 (3)	−0.010 (3)	0.019 (3)
C9	0.073 (3)	0.056 (2)	0.075 (3)	−0.005 (2)	0.004 (2)	0.007 (2)
C10	0.073 (3)	0.054 (2)	0.063 (3)	−0.012 (2)	0.008 (2)	0.0020 (19)
C11	0.063 (2)	0.057 (2)	0.059 (2)	−0.0090 (19)	0.000 (2)	−0.001 (2)
C12	0.057 (2)	0.053 (2)	0.047 (2)	−0.0052 (17)	−0.0048 (17)	0.0039 (17)
C13	0.048 (2)	0.057 (2)	0.058 (2)	−0.0037 (18)	0.0050 (18)	−0.0016 (18)
C14	0.047 (2)	0.071 (3)	0.080 (3)	0.001 (2)	0.010 (2)	−0.001 (2)
C15	0.050 (2)	0.069 (3)	0.081 (3)	0.011 (2)	−0.001 (2)	−0.007 (2)
C16	0.064 (2)	0.052 (2)	0.061 (2)	0.0017 (19)	−0.011 (2)	−0.0077 (19)
C17	0.075 (3)	0.064 (3)	0.072 (3)	0.004 (2)	−0.011 (2)	0.008 (2)
C18	0.106 (4)	0.068 (3)	0.069 (3)	0.001 (3)	−0.005 (3)	0.019 (2)
C19	0.091 (3)	0.079 (3)	0.064 (3)	−0.003 (3)	0.013 (3)	0.013 (2)
C20	0.070 (3)	0.067 (3)	0.060 (3)	0.003 (2)	0.012 (2)	0.006 (2)
C21	0.054 (2)	0.048 (2)	0.053 (2)	−0.0029 (17)	0.0006 (17)	−0.0022 (17)
C22	0.050 (2)	0.048 (2)	0.050 (2)	−0.0012 (16)	−0.0007 (16)	−0.0053 (16)
C23	0.0459 (18)	0.047 (2)	0.053 (2)	0.0008 (16)	0.0070 (16)	−0.0009 (17)
C24	0.0447 (17)	0.0476 (19)	0.0386 (18)	−0.0071 (15)	0.0062 (15)	−0.0008 (15)
C25	0.123 (5)	0.123 (5)	0.060 (3)	0.036 (4)	−0.021 (3)	−0.018 (3)
C26	0.119 (3)	0.127 (5)	0.093 (4)	0.025 (3)	−0.021 (2)	−0.002 (3)
C27	0.131 (3)	0.117 (3)	0.093 (3)	0.036 (3)	−0.002 (2)	−0.010 (3)
C28	0.109 (3)	0.105 (3)	0.0665 (12)	0.033 (3)	0.009 (2)	0.005 (2)
C29	0.126 (3)	0.119 (3)	0.072 (3)	0.026 (3)	0.022 (3)	0.007 (3)
C30	0.137 (4)	0.138 (4)	0.109 (4)	0.021 (3)	0.032 (3)	0.022 (3)
C31	0.123 (4)	0.118 (4)	0.103 (3)	0.020 (3)	0.025 (3)	0.017 (3)
C32	0.126 (4)	0.108 (4)	0.076 (3)	0.045 (4)	0.043 (3)	0.030 (3)
C33	0.116 (4)	0.105 (4)	0.0553 (15)	0.061 (3)	0.026 (2)	0.022 (2)
C34	0.105 (3)	0.088 (3)	0.050 (3)	0.045 (3)	0.000 (2)	0.002 (2)
C35	0.080 (3)	0.072 (3)	0.042 (2)	0.021 (2)	0.006 (2)	0.0029 (19)
C36	0.0509 (19)	0.048 (2)	0.047 (2)	0.0081 (16)	0.0000 (16)	−0.0023 (16)
C37	0.098 (7)	0.069 (6)	0.091 (7)	−0.002 (5)	−0.009 (6)	0.005 (5)
C38	0.104 (4)	0.079 (4)	0.081 (4)	0.007 (3)	−0.013 (4)	0.012 (3)
C39	0.091 (7)	0.082 (6)	0.056 (5)	0.006 (5)	−0.024 (5)	0.014 (5)
N10	0.071 (4)	0.037 (3)	0.057 (4)	0.015 (3)	−0.020 (3)	0.011 (3)
O4	0.105 (4)	0.095 (4)	0.078 (3)	−0.016 (3)	−0.027 (3)	0.037 (3)
C40	0.116 (8)	0.080 (7)	0.063 (6)	0.031 (6)	−0.002 (6)	−0.007 (5)
C41	0.162 (12)	0.139 (11)	0.189 (15)	0.098 (9)	0.052 (11)	0.082 (10)
C42	0.157 (12)	0.105 (10)	0.112 (11)	−0.033 (9)	0.004 (10)	−0.007 (8)
N11	0.079 (4)	0.050 (4)	0.041 (3)	0.008 (3)	0.015 (3)	0.004 (3)
O5	0.222 (10)	0.130 (6)	0.054 (4)	−0.029 (7)	−0.002 (5)	0.058 (4)
O6	0.115 (5)	0.103 (5)	0.055 (3)	0.005 (4)	0.030 (3)	0.049 (3)
C43	0.086 (6)	0.071 (6)	0.084 (7)	−0.016 (5)	−0.003 (5)	0.017 (5)
C44	0.092 (7)	0.084 (7)	0.076 (6)	−0.011 (5)	0.004 (5)	0.033 (5)
C45	0.082 (4)	0.104 (5)	0.082 (4)	0.000 (4)	0.014 (3)	0.004 (4)
N12	0.049 (3)	0.103 (5)	0.038 (3)	0.001 (4)	0.000 (3)	0.029 (3)
O7	0.086 (3)	0.049 (2)	0.038 (2)	−0.033 (2)	−0.010 (2)	0.0095 (19)
C46	0.081 (6)	0.048 (5)	0.096 (7)	−0.031 (4)	0.010 (5)	0.001 (5)
C47	0.078 (6)	0.060 (5)	0.076 (6)	−0.007 (4)	−0.024 (5)	−0.009 (4)
C48	0.101 (6)	0.051 (4)	0.025 (3)	−0.008 (4)	−0.005 (4)	0.007 (3)
N13	0.058 (3)	0.031 (3)	0.050 (4)	−0.010 (3)	0.002 (3)	−0.009 (2)

### Geometric parameters (Å, º)

**Table:**

Ag1—N5	2.282 (3)	C18—C19	1.377 (7)
Ag1—S3^i^	2.4869 (10)	C18—H18A	0.9300
Ag1—S1	2.5039 (10)	C19—C20	1.378 (6)
Ag1—Ag2	2.9329 (5)	C19—H19A	0.9300
Ag1—Ag3	2.9769 (6)	C20—C21	1.409 (6)
Ag2—N8^i^	2.294 (3)	C20—H20A	0.9300
Ag2—S1^i^	2.4699 (10)	C21—C22	1.428 (5)
Ag2—S2	2.4917 (11)	C22—C23	1.459 (5)
Ag2—Ag3^i^	3.0931 (5)	C23—H23A	0.9300
Ag2—Ag3	3.1835 (5)	C25—C34	1.364 (8)
Ag3—N2	2.282 (3)	C25—C26	1.438 (8)
Ag3—S2	2.4741 (11)	C26—C27	1.330 (10)
Ag3—S3	2.5020 (10)	C26—H26A	0.9300
Ag3—Ag2^i^	3.0931 (5)	C27—C28	1.350 (9)
S1—C12	1.759 (4)	C27—H27A	0.9300
S1—Ag2^i^	2.4699 (10)	C28—C33	1.436 (7)
S2—C24	1.755 (4)	C28—C29	1.438 (9)
S3—C36	1.762 (4)	C29—C30	1.248 (10)
S3—Ag1^i^	2.4869 (10)	C29—H29A	0.9300
N1—C11	1.264 (5)	C30—C31	1.438 (10)
N1—N2	1.384 (5)	C30—H30A	0.9300
N2—C12	1.293 (5)	C31—C32	1.363 (9)
N3—C12	1.335 (6)	C31—H31A	0.9300
N3—H3A	0.8600	C32—C33	1.403 (9)
N3—H3B	0.8600	C32—H32A	0.9300
N4—C23	1.273 (5)	C33—C34	1.480 (8)
N4—N5	1.390 (4)	C34—C35	1.454 (6)
N5—C24	1.310 (5)	C35—H35A	0.9300
N6—C24	1.330 (5)	C37—N10	1.432 (12)
N6—H6A	0.8600	C37—H37A	0.9600
N6—H6B	0.8600	C37—H37B	0.9600
N7—C35	1.270 (6)	C37—H37C	0.9600
N7—N8	1.410 (5)	C38—N10	1.516 (12)
N8—C36	1.302 (5)	C38—H38A	0.9600
N8—Ag2^i^	2.294 (3)	C38—H38B	0.9600
N9—C36	1.315 (5)	C38—H38C	0.9600
N9—H9A	0.8600	C39—O4	1.250 (12)
N9—H9B	0.8600	C39—N10	1.302 (11)
O1—C1	1.354 (6)	C39—H39A	0.9300
O1—H1B	0.8200	C40—N11	1.406 (11)
O2—C13	1.345 (5)	C40—H40A	0.9600
O2—H2B	0.8200	C40—H40B	0.9600
O3—C25	1.361 (8)	C40—H40C	0.9600
O3—H3C	0.8200	C41—N11	1.159 (16)
C1—C10	1.384 (6)	C41—H41A	0.9600
C1—C2	1.411 (7)	C41—H41B	0.9600
C2—C3	1.369 (7)	C41—H41C	0.9600
C2—H2A	0.9300	C42—O5	1.052 (16)
C3—C4	1.377 (7)	C42—N11	1.372 (16)
C3—H3D	0.9300	C42—H42A	0.9300
C4—C9	1.419 (6)	O6—C45	1.215 (14)
C4—C5	1.461 (8)	C43—N12	1.425 (13)
C5—C6	1.340 (9)	C43—H43A	0.9600
C5—H5A	0.9300	C43—H43B	0.9600
C6—C7	1.352 (8)	C43—H43C	0.9600
C6—H6C	0.9300	C44—N12	1.415 (13)
C7—C8	1.377 (8)	C44—H44A	0.9600
C7—H7B	0.9300	C44—H44B	0.9600
C8—C9	1.422 (7)	C44—H44C	0.9600
C8—H8B	0.9300	C45—N12	1.320 (13)
C9—C10	1.428 (6)	C45—H45A	0.9300
C10—C11	1.463 (6)	O7—C48	1.171 (10)
C11—H11A	0.9300	C46—N13	1.463 (10)
C13—C22	1.393 (5)	C46—H46A	0.9600
C13—C14	1.404 (6)	C46—H46B	0.9600
C14—C15	1.343 (7)	C46—H46C	0.9600
C14—H14A	0.9300	C47—N13	1.428 (11)
C15—C16	1.407 (6)	C47—H47A	0.9600
C15—H15A	0.9300	C47—H47B	0.9600
C16—C17	1.423 (6)	C47—H47C	0.9600
C16—C21	1.431 (6)	C48—N13	1.338 (10)
C17—C18	1.334 (7)	C48—H48A	0.9300
C17—H17A	0.9300		
			
N5—Ag1—S3^i^	123.04 (8)	C18—C19—C20	121.5 (5)
N5—Ag1—S1	116.61 (8)	C18—C19—H19A	119.3
S3^i^—Ag1—S1	114.45 (3)	C20—C19—H19A	119.3
N5—Ag1—Ag2	85.53 (8)	C19—C20—C21	120.8 (4)
S3^i^—Ag1—Ag2	78.36 (2)	C19—C20—H20A	119.6
S1—Ag1—Ag2	132.17 (3)	C21—C20—H20A	119.6
N5—Ag1—Ag3	82.03 (8)	C20—C21—C22	124.4 (4)
S3^i^—Ag1—Ag3	134.25 (3)	C20—C21—C16	117.0 (4)
S1—Ag1—Ag3	76.10 (2)	C22—C21—C16	118.6 (4)
Ag2—Ag1—Ag3	65.183 (11)	C13—C22—C21	119.4 (3)
N8^i^—Ag2—S1^i^	115.91 (9)	C13—C22—C23	119.8 (3)
N8^i^—Ag2—S2	116.15 (9)	C21—C22—C23	120.8 (3)
S1^i^—Ag2—S2	119.55 (4)	N4—C23—C22	123.1 (3)
N8^i^—Ag2—Ag1	81.60 (8)	N4—C23—H23A	118.5
S1^i^—Ag2—Ag1	136.94 (3)	C22—C23—H23A	118.5
S2—Ag2—Ag1	79.02 (2)	N5—C24—N6	124.4 (3)
N8^i^—Ag2—Ag3^i^	83.84 (8)	N5—C24—S2	120.5 (3)
S1^i^—Ag2—Ag3^i^	74.37 (2)	N6—C24—S2	115.1 (3)
S2—Ag2—Ag3^i^	139.12 (3)	O3—C25—C34	121.8 (5)
Ag1—Ag2—Ag3^i^	68.654 (13)	O3—C25—C26	118.3 (6)
N8^i^—Ag2—Ag3	138.11 (8)	C34—C25—C26	119.9 (6)
S1^i^—Ag2—Ag3	102.31 (3)	C27—C26—C25	118.0 (7)
S2—Ag2—Ag3	49.88 (2)	C27—C26—H26A	121.0
Ag1—Ag2—Ag3	58.076 (12)	C25—C26—H26A	121.0
Ag3^i^—Ag2—Ag3	90.886 (14)	C26—C27—C28	127.3 (7)
N2—Ag3—S2	123.17 (9)	C26—C27—H27A	116.3
N2—Ag3—S3	109.62 (9)	C28—C27—H27A	116.3
S2—Ag3—S3	118.61 (3)	C27—C28—C33	117.0 (6)
N2—Ag3—Ag1	87.24 (8)	C27—C28—C29	125.3 (6)
S2—Ag3—Ag1	78.42 (3)	C33—C28—C29	117.7 (6)
S3—Ag3—Ag1	136.20 (3)	C30—C29—C28	122.0 (7)
N2—Ag3—Ag2^i^	80.83 (8)	C30—C29—H29A	119.0
S2—Ag3—Ag2^i^	137.87 (3)	C28—C29—H29A	119.0
S3—Ag3—Ag2^i^	75.09 (2)	C29—C30—C31	122.4 (8)
Ag1—Ag3—Ag2^i^	67.966 (11)	C29—C30—H30A	118.8
N2—Ag3—Ag2	143.62 (8)	C31—C30—H30A	118.8
S2—Ag3—Ag2	50.37 (2)	C32—C31—C30	118.7 (7)
S3—Ag3—Ag2	101.20 (3)	C32—C31—H31A	120.7
Ag1—Ag3—Ag2	56.741 (12)	C30—C31—H31A	120.7
Ag2^i^—Ag3—Ag2	89.114 (14)	C31—C32—C33	120.9 (6)
C12—S1—Ag2^i^	104.32 (14)	C31—C32—H32A	119.5
C12—S1—Ag1	108.93 (14)	C33—C32—H32A	119.5
Ag2^i^—S1—Ag1	86.06 (3)	C32—C33—C28	118.1 (5)
C24—S2—Ag3	106.25 (12)	C32—C33—C34	124.0 (5)
C24—S2—Ag2	104.41 (12)	C28—C33—C34	117.9 (6)
Ag3—S2—Ag2	79.74 (3)	C25—C34—C35	121.0 (5)
C36—S3—Ag1^i^	107.32 (13)	C25—C34—C33	119.8 (5)
C36—S3—Ag3	101.81 (12)	C35—C34—C33	119.1 (5)
Ag1^i^—S3—Ag3	85.95 (3)	N7—C35—C34	121.9 (5)
C11—N1—N2	115.3 (3)	N7—C35—H35A	119.1
C12—N2—N1	114.6 (3)	C34—C35—H35A	119.1
C12—N2—Ag3	121.3 (3)	N8—C36—N9	124.8 (4)
N1—N2—Ag3	124.1 (2)	N8—C36—S3	119.2 (3)
C12—N3—H3A	120.0	N9—C36—S3	116.0 (3)
C12—N3—H3B	120.0	N10—C37—H37A	109.5
H3A—N3—H3B	120.0	N10—C37—H37B	109.5
C23—N4—N5	115.3 (3)	H37A—C37—H37B	109.5
C24—N5—N4	114.3 (3)	N10—C37—H37C	109.5
C24—N5—Ag1	122.4 (2)	H37A—C37—H37C	109.5
N4—N5—Ag1	122.9 (2)	H37B—C37—H37C	109.5
C24—N6—H6A	120.0	N10—C38—H38A	109.5
C24—N6—H6B	120.0	N10—C38—H38B	109.5
H6A—N6—H6B	120.0	H38A—C38—H38B	109.5
C35—N7—N8	114.1 (4)	N10—C38—H38C	109.5
C36—N8—N7	114.2 (3)	H38A—C38—H38C	109.5
C36—N8—Ag2^i^	121.0 (3)	H38B—C38—H38C	109.5
N7—N8—Ag2^i^	120.3 (2)	O4—C39—N10	122.9 (9)
C36—N9—H9A	120.0	O4—C39—H39A	118.5
C36—N9—H9B	120.0	N10—C39—H39A	118.5
H9A—N9—H9B	120.0	C39—N10—C37	120.2 (8)
C1—O1—H1B	109.5	C39—N10—C38	118.4 (8)
C13—O2—H2B	109.5	C37—N10—C38	121.3 (7)
C25—O3—H3C	109.5	N11—C40—H40A	109.5
O1—C1—C10	122.3 (4)	N11—C40—H40B	109.5
O1—C1—C2	116.5 (4)	H40A—C40—H40B	109.5
C10—C1—C2	121.1 (5)	N11—C40—H40C	109.5
C3—C2—C1	119.2 (5)	H40A—C40—H40C	109.5
C3—C2—H2A	120.4	H40B—C40—H40C	109.5
C1—C2—H2A	120.4	N11—C41—H41A	109.5
C2—C3—C4	121.7 (5)	N11—C41—H41B	109.5
C2—C3—H3D	119.1	H41A—C41—H41B	109.5
C4—C3—H3D	119.1	N11—C41—H41C	109.5
C3—C4—C9	120.1 (4)	H41A—C41—H41C	109.5
C3—C4—C5	121.9 (5)	H41B—C41—H41C	109.5
C9—C4—C5	118.0 (5)	O5—C42—N11	137.7 (15)
C6—C5—C4	121.0 (5)	O5—C42—H42A	111.2
C6—C5—H5A	119.5	N11—C42—H42A	111.2
C4—C5—H5A	119.5	C41—N11—C42	119.4 (11)
C5—C6—C7	121.3 (6)	C41—N11—C40	113.8 (10)
C5—C6—H6C	119.3	C42—N11—C40	126.8 (9)
C7—C6—H6C	119.3	N12—C43—H43A	109.5
C6—C7—C8	120.7 (6)	N12—C43—H43B	109.5
C6—C7—H7B	119.6	H43A—C43—H43B	109.5
C8—C7—H7B	119.6	N12—C43—H43C	109.5
C7—C8—C9	121.8 (5)	H43A—C43—H43C	109.5
C7—C8—H8B	119.1	H43B—C43—H43C	109.5
C9—C8—H8B	119.1	N12—C44—H44A	109.5
C4—C9—C8	117.1 (4)	N12—C44—H44B	109.5
C4—C9—C10	118.7 (4)	H44A—C44—H44B	109.5
C8—C9—C10	124.2 (4)	N12—C44—H44C	109.5
C1—C10—C9	119.1 (4)	H44A—C44—H44C	109.5
C1—C10—C11	119.9 (4)	H44B—C44—H44C	109.5
C9—C10—C11	120.9 (4)	O6—C45—N12	123.7 (11)
N1—C11—C10	123.2 (4)	O6—C45—H45A	118.1
N1—C11—H11A	118.4	N12—C45—H45A	118.1
C10—C11—H11A	118.4	C45—N12—C44	120.4 (9)
N2—C12—N3	124.7 (4)	C45—N12—C43	122.3 (9)
N2—C12—S1	120.5 (3)	C44—N12—C43	117.2 (7)
N3—C12—S1	114.8 (3)	N13—C46—H46A	109.5
O2—C13—C22	122.2 (3)	N13—C46—H46B	109.5
O2—C13—C14	117.3 (4)	H46A—C46—H46B	109.5
C22—C13—C14	120.5 (4)	N13—C46—H46C	109.5
C15—C14—C13	121.0 (4)	H46A—C46—H46C	109.5
C15—C14—H14A	119.5	H46B—C46—H46C	109.5
C13—C14—H14A	119.5	N13—C47—H47A	109.5
C14—C15—C16	121.3 (4)	N13—C47—H47B	109.5
C14—C15—H15A	119.4	H47A—C47—H47B	109.5
C16—C15—H15A	119.4	N13—C47—H47C	109.5
C15—C16—C17	121.5 (4)	H47A—C47—H47C	109.5
C15—C16—C21	119.2 (4)	H47B—C47—H47C	109.5
C17—C16—C21	119.3 (4)	O7—C48—N13	127.4 (7)
C18—C17—C16	121.3 (4)	O7—C48—H48A	116.3
C18—C17—H17A	119.3	N13—C48—H48A	116.3
C16—C17—H17A	119.3	C48—N13—C47	118.8 (6)
C17—C18—C19	120.1 (5)	C48—N13—C46	122.1 (7)
C17—C18—H18A	119.9	C47—N13—C46	119.1 (7)
C19—C18—H18A	119.9		

Symmetry code: (i) −*x*+1/2, −*y*+3/2, −*z*+1.

### Hydrogen-bond geometry (Å, º)

**Table:**

*D*—H···*A*	*D*—H	H···*A*	*D*···*A*	*D*—H···*A*
N3—H3*B*···O4	0.86	1.98	2.835 (8)	175
N6—H6*B*···O6^i^	0.86	2.00	2.845 (7)	166
N9—H9*A*···O5^ii^	0.86	2.31	3.049 (8)	145
N9—H9*B*···O7	0.86	2.02	2.870 (6)	172
O1—H1*B*···N1	0.82	1.86	2.588 (5)	147
O2—H2*B*···N4	0.82	1.85	2.583 (4)	148
O3—H3*C*···N7	0.82	1.86	2.587 (5)	147

Symmetry codes: (i) −*x*+1/2, −*y*+3/2, −*z*+1; (ii) −*x*+1/2, *y*−1/2, −*z*+1/2.
